# Anemia is a common but neglected complication of adjuvant chemotherapy for early breast cancer

**DOI:** 10.3747/co.2007.156

**Published:** 2007-12

**Authors:** A. Goldrick, I.A. Olivotto, C.S. Alexander, C.H. Speers, J. Barnett, S.J. Allan, P.T. Truong

**Affiliations:** * Breast Cancer Outcomes Unit, BC Cancer Agency, Vancouver, British Columbia; † Systemic Therapy Program, BC Cancer Agency, Vancouver Island Centre, Victoria, British Columbia; ‡ Liverpool Hospital, Sydney, New South Wales, Australia.; § Radiation Therapy Program, BC Cancer Agency, Vancouver Island Centre, Victoria, British Columbia.; || University of British Columbia, Vancouver, British Columbia

**Keywords:** Anemia, breast cancer, chemotherapy, transfusion, epoetin

## Abstract

In this study, we set out to determine the frequency and severity of anemia and the corrective interventions used during adjuvant chemotherapy for breast cancer.

We conducted a retrospective electronic chart review of 702 patients who received adjuvant breast cancer chemotherapy at four BC Cancer Agency centres in 2002 and 2003. For these patients, we recorded the initial hemoglobin reading and the date of the first hemoglobin reading in the ranges 110–119 g/L, 100–109 g/L, 90–99 g/L, and <90 g/L. We also recorded any discussion about, or delivery of, interventions for anemia [transfusion, epoetin (epo) or both].

Median age of the study population was 51 years, and it varied with chemotherapy type. Among the patients, 12% had a hemoglobin reading <120 g/L before the start of chemotherapy. Overall, the proportion of patients with at least one hemoglobin reading <120 g/L was 78%; <110 g/L, 54%; <100 g/L, 31%; and <90 g/L, 14%. Depending on chemotherapy type, a hemoglobin reading <100 g/L occurred in 5% to 54% of patients. Intervention rates increased as hemoglobin declined. For 99 patients with a hemoglobin reading <90 g/L, a discussion of anemia was documented in the treatment chart in 49% of cases, a transfusion was delivered in 23%, epo was used in 11%, and transfusion and epo were both delivered in 5%.

Anemia was relatively common and varied with chemotherapy type. Documentation of a discussion of anemia occurred in fewer than 20% of the patients with a hemoglobin reading of 90–99 g/L and in only half the patients with a hemoglobin reading <90 g/L. Intervention rates were low at hemoglobin readings for which randomized trials have shown that intervention can improve quality of life.

## 1. INTRODUCTION

Multi-agent chemotherapy has a spectrum of side effects, including anemia and fatigue. The severity of anemia and fatigue vary with chemotherapy, disease type, age, and other factors 1–5. Patients who develop anemia during cancer therapy report more fatigue and poorer quality of life 6,7. The treatment of anemia with growth factor support has been associated with reduced fatigue and improved quality of life 8–14.

Physicians treating patients with adjuvant chemotherapy for breast cancer often focus on potentially life-threatening toxicities such as febrile neutropenia or toxicities that require immediate symptomatic intervention or dose reductions, such as mucositis, diarrhea, or neuropathy. Fatigue and anemia are often more insidious, but their impact on quality of life is an important concern for patients with cancer 15. The frequency and severity of anemia associated with many commonly used adjuvant chemotherapy regimens for early-stage breast cancer have not been well documented. The present study reports the frequency and severity of anemia that developed during adjuvant chemotherapy for breast cancer in patients treated at four Canadian regional cancer centres, and the interventions for anemia that were delivered during that chemotherapy.

## 2. PATIENTS AND METHODS

### 2.1 Setting and Patients

The BC Cancer Agency (bcca) manages the budget for all anti-neoplastic drugs in the Canadian province of British Columbia. We used a provincial pharmacy database to identify women with breast cancer who had received adjuvant chemotherapy in 2002 and 2003 at any of four bcca regional cancer centres (designated a, b, c, and d). Patients were excluded if the chemotherapy had been administered for metastatic disease, if the patient had had prior chemotherapy, if fewer than 3 cycles of chemotherapy had been delivered, or if the chemotherapy had been received outside of the four regional cancer centres.

### 2.2 Data Collection

Using the bcca electronic health record, a single health records administrator (CSA) retrospectively reviewed prospectively-collected chart data. Dates of diagnosis, first and last adjuvant chemotherapy, pre-chemotherapy hemoglobin reading, and the first hemoglobin readings <120 g/L, <110 g/L, <100 g/L, and <90 g/L were recorded, as were patient age at diagnosis, disease stage, adjuvant chemotherapy type or types, treating centre, and name of the attending medical oncologist. In addition, the data abstractor reviewed the narrative notes, correspondence, and ancillary information in the chart and recorded any discussion of anemia or intervention for anemia (transfusion, use of growth factor support). A patient who received epoetin (epo) or a transfusion was considered to have had a discussion of anemia.

Dates of interventions for anemia—whether a discussion, a transfusion, or delivery of epo—were recorded and attributed to the interval of the hemoglobin reading most immediately preceding the date of intervention. The magnitude of fatigue and of effects of chemotherapy on quality of life were not recorded prospectively and could not be reliably deduced in retrospect, and so no effort was made to relate hemoglobin reading or anemia intervention to quality of life.

### 2.3 Adjuvant Therapies Evaluated

Adjuvant chemotherapy included these regimens:

- ac: doxorubicin + cyclophosphamide × 4 cycles 16
- cef: cyclophosphamide + epirubicin + 5-fluorouracil × 6 cycles 17
- ac-t/d: ac × 4 cycles, followed by paclitaxel or docetaxel × 4 cycles 18
- caf: cyclophosphamide + doxorubicin + 5-fluorouracil
- cmf: cyclophosphamide + methotrexate + 5-fluorouracil 16
- fec100: 5-fluorouracil + epirubicin 100 mg/m^2^ every 3 weeks intravenously + cyclophosphamide 19

The most frequently used chemotherapy regimen was ac. Because ac was not expected to cause much anemia 16, only a random sample of ac cases was included in the analysis. Because the number of patients treated varied between the four cancer centres, our analysis included all patients receiving ac at the smallest centre, a 50% random sample from each of the two intermediate-size centres, and a 33% random sample from the largest centre. All patients receiving any of the other chemotherapy regimens were included.

### 2.4 Statistical Analyses

All data collected were entered into a searchable database. Descriptive statistics were generated. A patient was counted only the first time she showed a hemoglobin reading in each of the following ranges: 110–119 g/L, 100–109 g/L, 90–99 g/L, and <90 g/L. If a patient had a progressively declining hemoglobin reading, she would have contributed data once at each hemoglobin interval for which she had a reading in the relevant range.

### 2.5 Declarations

Our study was sponsored by Ortho Biotech Canada and was approved by the Human Research Ethics Board of the bcca and the University of British Columbia.

The sponsor provided funding support only and had no direct role in data collection, analysis, or interpretation. The right to approve or disapprove publication of the manuscript was held by the investigators only.

## 3. RESULTS

From among an initial 1092 patients identified in the pharmacy database as having received adjuvant chemotherapy for breast cancer in 2002 and 2003, 159 patients were excluded because of prior breast cancer (*n* = 59), prior chemotherapy (*n* = 11), treatment outside one of the four bcca centres (*n* = 30), fewer than 3 cycles of chemotherapy (*n* = 7), or treatment received for recurrent or metastatic disease (*n* = 52). Of the remaining 933 cases that were eligible for analysis, 459 had received ac, and 228 of those were randomly selected for inclusion, yielding a total analysis sample of 702 patients. Table I summarizes the study cohort’s pertinent demographic and treatment characteristics.

The use of chemotherapy was similar between centres, except for somewhat greater use of ac or cmf chemotherapy at centre c, in part because older women formed a greater proportion of the patients treated at that centre (Table II).

**FIGURE 1:**
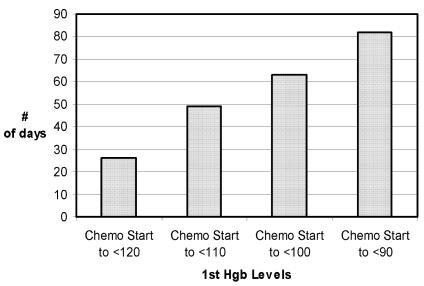
Median duration between the start of chemotherapy and first hemoglobin (Hgb) reading at each level (patients with a pre-chemotherapy Hgb < 120 g/L were excluded).

Anemia (hemoglobin < 100 g/L) was associated with chemotherapy type and patient age (Table III). The severity of the anemia increased with the duration of chemotherapy (Figure 1). When corrected for the type of chemotherapy used, the proportion of patients developing a hemoglobin reading of <100 g/L was similar between the treating centres (data not shown).

### 3.1 Interventions for Anemia

**FIGURE 2:**
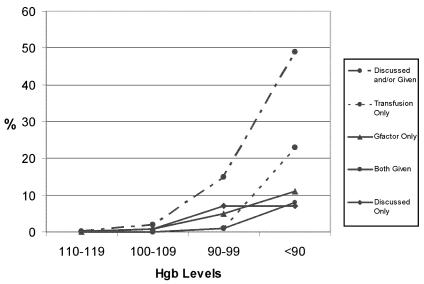
The proportion of patients having a discussion of, or intervention for, anemia increased at lower hemoglobin (Hgb) levels.

Figure 2 shows the proportions of patients with hemoglobin readings in the four study ranges for whom a discussion of anemia was recorded in the narrative notes of the patient’s bcca chart. It also shows the proportions of patients whose chart showed evidence that a transfusion or epo, or both, were prescribed. Evidence that a discussion of anemia had occurred during an outpatient visit to a bcca centre was found for 15% of patients with a hemoglobin reading in the range 90–99 g/L (*n* = 28) and for 49% of patients with a hemoglobin reading of <90 g/L (*n* = 49). The respective proportions of those patients receiving a transfusion were 0.93% (*n* = 2) and 23% (*n* = 23), receiving epo were 5% (*n* = 10) and 11% (*n* = 11), and receiving both were 0.93% (*n* = 2) and 8% (*n* = 8).

### 3.2 Interventions for Anemia Among Patients Receiving CEF

**FIGURE 3:**
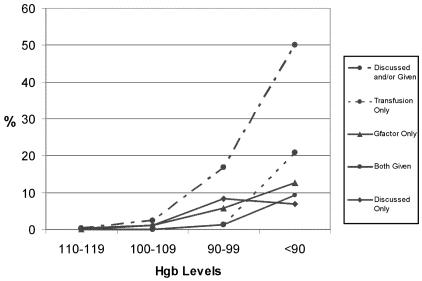
Discussion of or treatment for anemia in patients receiving cef (cyclophosphamide + epirubicin + 5-fluorouracil) chemotherapy (*n* = 325), by hemoglobin (Hgb) level.

**FIGURE 4:**
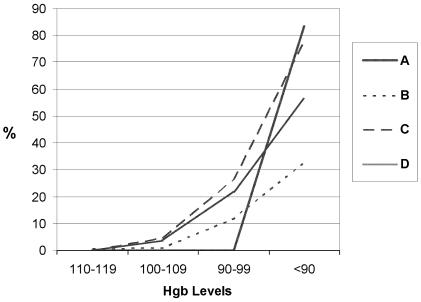
Rates of discussion of or intervention for anemia in patients receiving cef (cyclophosphamide + epirubicin + 5-fluorouracil) chemotherapy (*n* = 325), by cancer centre. Hgb = hemoglobin; A, B, C, D = the four study centres.

Patients receiving cef chemotherapy most consistently developed anemia (hemoglobin < 100 g/L) and severe anemia (hemoglobin < 90 g/L). Figure 3 shows the proportion of patients receiving cef whose chart showed evidence of a discussion or intervention for anemia at the various study hemoglobin ranges. Just 50% of study patients receiving cef had evidence of a discussion of anemia in their chart when their hemoglobin reading fell below 90 g/L. The intervention rates were much lower than the discussion rates shown in Figure 3. Figure 4 shows, for each bcca centre, the proportion of patients receiving cef who had any intervention (discussion, transfusion, epo, or a combination) at the various study hemoglobin ranges. The intervention rate, including discussion of or an intervention for anemia, varied between the four centres.

## 4. DISCUSSION

Our study demonstrates that anemia is a common side effect of adjuvant chemotherapy for breast cancer. Overall, 31% of patients receiving multi-agent chemotherapy for early-stage breast cancer at a bcca centre showed at least 1 hemoglobin reading below 100 g/L, and 14% showed at least 1 hemoglobin value below 90 g/L. For cef and ac-t/d chemotherapy, the proportions developing anemia (hemoglobin < 100 g/L) were 54% and 27% respectively, and the proportions developing severe anemia (hemoglobin < 90 g/L) were 27% and 7% respectively.

Few data reporting anemia rates with various adjuvant chemotherapy regimens for breast cancer are available for comparison with these results. A retrospective series of patients treated in community practice with ac chemotherapy reported that 17% of patients developed anemia (hemoglobin < 100 g/L) 20, a rate that is substantially higher than the 5% observed in the current study. In a series of 310 patients with stage ii and iii breast cancer treated with adjuvant ac chemotherapy at eight U.S. centres, 40% showed a drop in hemoglobin reading to less than 100 g/L during the course of treatment 21.

Because the frequency and severity of anemia varied with chemotherapy type and was most severe among patients receiving cef 17 chemotherapy, we repeated our analysis and cross-centre comparisons for patients receiving cef chemotherapy (*n* = 325). Even when patients were receiving cef, an adjuvant program known to be associated with fatigue and anemia, the charts of only 50% contained a recorded discussion of anemia when a hemoglobin reading of <90 g/L was recorded. Documentation that a discussion had occurred varied by treatment centre, ranging from 32% to 83%.

The purpose of the present study was not to determine who should have an intervention for anemia or whether intervention improves quality of life. The 2002 American Society of Clinical Oncology/American Society of Hematology guideline on the use of epo in patients with chemotherapy-associated anemia recommends treatment at a hemoglobin reading of <100 g/L 8. This grade b recommendation is based on level ii evidence. In patients with less severe anemia (hemoglobin 100–120 g/L), the guideline recommends that treatment decisions be guided by an evaluation of the clinical manifestations of anemia, including its effect on the patient’s functional capacity and quality of life (grade c recommendation) 8.

Anemia is a relatively neglected side effect of adjuvant chemotherapy. In the present study, the treatment records of only 15% of patients with a hemoglobin reading of 90–99 g/L and 49% of those with a hemoglobin reading <90 g/L contained evidence that anemia had at least been discussed. The therapeutic intervention rate (transfusion or epo, or both) was even lower: 6.5% (*n* = 14) for patients with a hemoglobin reading of 90–99 g/L and 42% (*n* = 42) for patients with a hemoglobin reading of <90 g/L. Relatively low rates of intervention have also been reported in other studies 7,22. In the European Cancer Anemia Survey (ecas), which prospectively collected data on the incidence and prevalence of anemia in more than 15,000 cancer patients with various malignancies (including more than 3000 with breast cancer), 30% of the breast cancer patients were anemic (hemoglobin < 120 g/L) at survey enrolment 5. Anemia rates varied by tumour type, disease status, and cancer treatment status. In the ecas study, 19% of patients with breast cancer who had anemia received either a transfusion or epo, or a combination. Low hemoglobin levels correlated with poor performance status.

A number of reasons may account for the low rates observed in the current study of discussion of anemia and of intervention to correct the condition. Some patients may not have been symptomatic with a hemoglobin reading of <100 g/L or <90 g/L (an unlikely situation) 9,10. Some anemic patients may have been close to chemotherapy completion, and their physicians may have felt that they would recover without specific intervention. A discussion of anemia may have taken place, but may not have been recorded in the chart. (A retrospective evaluation of the latter circumstance is impossible, but the narrative notes were frequently lengthy, recording many other details of the patient’s cancer, treatment-related symptoms, and social circumstances.) The cost of treatment may have been another factor. The bcca funds all anti-neoplastic drugs in the province, including the cost to deliver chemotherapy, but it does not fund supportive medications, including anti-emetics, analgesics, or growth factor support. Supportive medications are the responsibility of the patient or of her extended health coverage provider. The likelihood that oncologists would discuss anemia and offer interventions if growth factor support were free of direct charge to the patient is unknown. Considerable debate exists about the ethics of discussing or withholding details about drug treatments that are not funded within a system of otherwise universal coverage 23,24.

Physicians and patients are increasingly reluctant to use transfusions for self-limited anemia, despite the reduction in risk of viral transmission with improved screening. Some physicians may be reluctant to use growth factor support because of concerns about compromising outcome during curative treatment, given that erythropoietin receptors have been reported to be present on some breast cancer cells 25,26, and endogenous erythropoietin may inhibit hypoxia-induced apoptosis of breast cancer cells 27. These complex issues all need to be considered before intervention for anemia and fatigue are recommended, but the consideration starts with recognizing that anemia and fatigue are a relatively common side effect of adjuvant chemotherapy.

Since this study was completed, the use of cef chemotherapy has declined considerably, and some bcca centres have adopted a practice of having the ambulatory care nurse review the patient’s hemoglobin reading before each cycle of chemotherapy, discuss quality-of-life issues with the patient, and highlight the level of fatigue, if present, to the attending oncologist if the hemoglobin reading is below 100 g/L. These measures have the potential to increase awareness of chemotherapy-related anemia as an adverse effect on the patient’s function and quality of life. Whether this approach will ultimately increase the rate of interventions to correct anemia and improve quality of life or compliance with planned treatment during adjuvant chemotherapy for breast cancer warrants prospective evaluation.

## 5. CONCLUSIONS

In breast cancer patients undergoing adjuvant chemotherapy, anemia was relatively common and varied with chemotherapy type. Documentation of a discussion of anemia occurred in fewer than 20% of the patients with a hemoglobin reading of 90–99 g/L and in only half the patients with a hemoglobin reading of <90 g/L. Intervention rates were low at hemoglobin readings for which randomized trials have shown that intervention can improve quality of life.

## Figures and Tables

**TABLE I:** Demographic, disease, and treatment characteristics of 702 patients receiving adjuvant chemotherapy for breast cancer at a BC Cancer Agency facility in 2002 or 2003

	Patients [*n* (%)]
Age (years)	
<50	234 (33.3)
50–59	289 (41.2)
60+	179 (25.5)
T stage	
TX	62 (8.8)
T1	259 (36.9)
T2	331 (47.2)
T3	42 (6)
T4	8 (1.1)
Nodal status	
N0	231 (32.9)
N1	352 (50.1)
N2	45 (6.4)
N3	18 (2.6)
Nx	56 (8)
Treatment centre	
a	70 (10)
b	230 (32.8)
c	158 (22.5)
d	244 (34.8)
Pre-chemotherapy hemoglobin (g/L)	
<120	83 (11.8)
≥120	619 (88.2)
Chemotherapy type	
ac	228 (32.5)
cef	325 (46.3)
ac-t/d	55 (7.8)
caf	46 (6.6)
cmf	31 (4.4)
fec100	17 (2.4)

ac = doxorubicin + cyclophosphamide; cef = cyclophosphamide + epirubicin + 5-fluorouracil; ac-t/d = doxorubicin + cyclophosphamide followed by paclitaxel or docetaxel; caf = cyclophosphamide + doxorubicin + 5-fluorouracil; cmf = cyclophosphamide + methotrexate + 5-fluorouracil; fec100 = 5-fluorouracil + epirubicin 100 mg/m^2^ every 3 weeks intravenously + cyclophosphamide.

**TABLE II:** Selected demographic and treatment characteristics for 702 patients receiving adjuvant chemotherapy for breast cancer

Centre	Patients (*n*)	Median age (years)	Age		cef [*n* (%)]	ac-t/d [*n* (%)]	ac or cmf [*n* (%)]	Hemoglobin <100 g/L [*n* (%)]
			<50 years [*n* (%)]	>60 years [*n* (%)]				
All	702	51	234 (33.3)	179 (25.5)	325 (46.3)	55 (7.8)	259 (36.9)	215 (30.6)
a	70	49	28 (40.0)	16 (22.9)	34 (48.6)	8 (11.4)	19 (27.1)	13 (18.6)
b	230	50	84 (36.5)	57 (24.8)	120 (52.2)	15 (6.5)	77 (33.5)	90 (39.1)
c	158	53	35 (22.2)	51 (32.3)	62 (39.2)	4 (2.5)	78 (49.4)	30 (9)
d	244	50	87 (35.7)	55 (22.5)	109 (44.7)	28 (11.5)	85 (34.8)	82 (33.6)

cef = cyclophosphamide + epirubicin + 5-fluorouracil; ac-t/d = doxorubicin + cyclophosphamide followed by paclitaxel or docetaxel; ac = doxorubicin + cyclophosphamide; cmf = cyclophosphamide + methotrexate + 5-fluorouracil.

**TABLE III:** Numbers and proportions of patients developing various severities of anemia during adjuvant chemotherapy for breast cancer

	Patients [*n* (%)]	Hemoglobin {g/L [*n* (%)]}			
		<120 (*n*=545)	<110 (*n*=380)	<100 (*n*=215)	<90 (*n*=99)
All 702					
Chemotherapy					
cef	325 (46.3)	319 (98.2)	272 (83.7)	175 (53.8)	86 (26.5)
ac-t/d	55 (7.8)	45 (81.8)	31 (56.4)	15 (27.3)	4 (7.3)
ac	228 (32.5)	120 (52.6)	44 (19.3)	11 (4.8)	3 (1.3)
cmf	31 (4.4)	16 (51.6)	8 (25.8)	3 (9.7)	3 (9.7)
caf	46 (6.6)	31 (67.4)	18 (39.1)	9 (19.6)	3 (6.5)
fec100	17 (2.4)	14 (82.4)	7 (41.2)	2 (11.8)	0
Centre					
a	70 (10)	53 (75.7)	30 (42.9)	13 (18.6)	6 (8.6)
b	230 (32.8)	185 (80.4)	145 (63.0)	90 (39.1)	42 (18.3)
c	158 (22.5)	115 (72.8)	142 (89.9)	82 (51.9)	42 (26.6)
s	244 (34.8)	192 (78.7)	63 (25.8)	30 (12.3)	9 (3.7)
Age (years)					
<50	234 (33.3)	200 (85.5)	143 (61.1)	78 (33.3)	37 (15.8)
≥50	468 (66.7)	345 (73.7)	237 (50.6)	137 (29.3)	62 (13.2)

cef = cyclophosphamide + epirubicin + 5-fluorouracil; ac-t/d = doxorubicin + cyclophosphamide followed by paclitaxel or docetaxel; ac = doxorubicin + cyclophosphamide; cmf = cyclophosphamide + methotrexate + 5-fluorouracil; caf = cyclophosphamide + doxorubicin + 5-fluorouracil; fec100 = 5-fluorouracil + epirubicin 100 mg/m^2^ every 3 weeks intravenously + cyclophosphamide.
